# A new hormonal marker for tracking rhino pregnancy

**DOI:** 10.1093/conphys/coag038

**Published:** 2026-06-17

**Authors:** Caroline E Terry

**Affiliations:** School of Biological Sciences, Washington State University, Pullman, WA, USA

Rhinos definitely do not make conservation easy. Black and white rhino pregnancies last well over a year—nearly twice as long as a typical human pregnancy—and have few outward signs of success or failure. Because of this, managers are often left guessing whether a calf is on the way. Strengthening population sizes of these rhino species is a major conservation focus, but this slow and unpredictable reproduction makes it difficult. To overcome this challenge, scientists and conservation managers are interested in improving how pregnancies are monitored to ensure positive outcomes. In a recent article, researchers from George Mason University (Arbogast *et al.*, 2026), along with managers and conservationists from 37 rhino-housing facilities across North America, tested a new hormonal measure to track rhino pregnancies.

Dehydroepiandosterone (DHEA) is a hormone involved in stress responses and the production of sex hormones like testosterone and estradiol. In other animals, such as horses, which are closely related to rhinos, DHEA levels rise and fall in consistent ways during pregnancy. Based on this, Drew Arbogast and team hypothesized that this hormone might be a useful indicator of rhino pregnancy. DHEA has been extracted and measured in other mammals, but not in rhinos, so the method needed to be fine-tuned specifically to rhino species. To do this, the team tested how well several different chemicals worked to extract DHEA from rhino blood serum—the clear liquid in blood that is left after removing blood cells. These chemicals make the serum separate into different layers, one of which contains all of the DHEA in the serum. After identifying which approach extracted the most DHEA, they used it to take monthly measurements in 40 black rhinos and 71 white rhinos for a whole year (Fig. 1).

**Figure 1:**
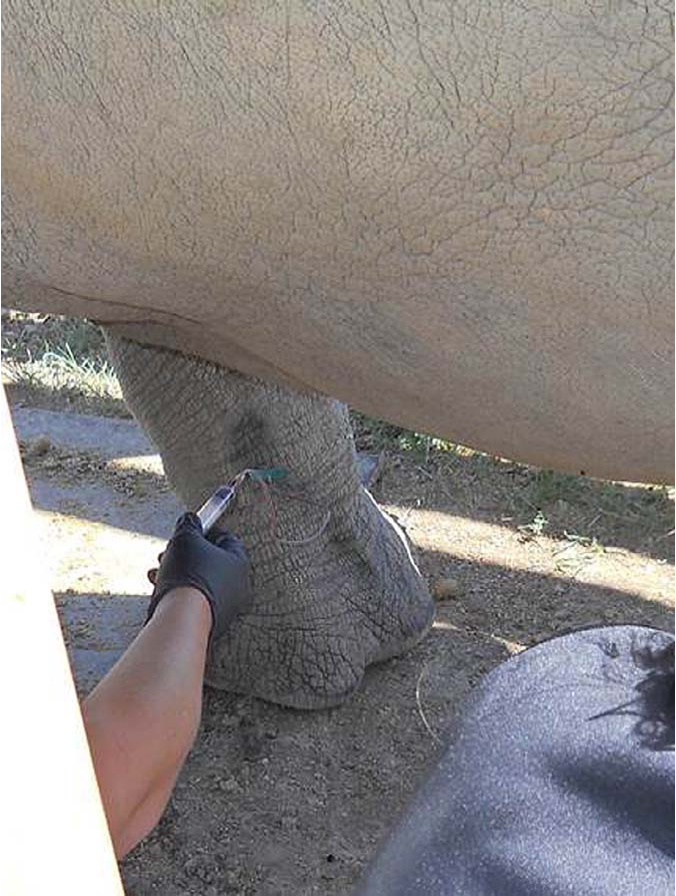
Voluntary blood draw to assess serial, point-in-time measures of a rhino’s physiological state. Image credit: Terri Roth.

These researchers found that in both rhino species, DHEA levels increased over a rhino’s life until 15 years of age, after which they begin to decline. This peak occurs around the time when rhinos are most actively breeding (8–17 years for black rhinos and 10–18 years for white rhinos), meaning that this hormone may be a good indicator of rhino age. DHEA levels also rose over the course of pregnancy compared to non-pregnant rhinos, especially during mid- to late pregnancy. Interestingly, this rise was not seen in one white rhino that miscarried during the study. This increase in DHEA levels in rhinos with healthy pregnancies suggests that this approach may help managers better monitor pregnancy status and foetal health in rhinos. Because rhino pregnancies last for over a year, the scientists could not use this hormone to track any pregnancies from start to finish, but they hope that this will be included in a future study.

Improving hormonal monitoring in captive rhinos can help scientists better understand challenges like irregular reproductive cycles and the progression of healthy pregnancies. Thanks to this research, scientists and rhino managers are now equipped with a better understanding of hormonal changes in rhinos during pregnancy. With further development, it may even enable non-invasive monitoring through faecal or urine samples, reducing stress on captive rhinos. This will ultimately contribute to more effective breeding programs and support the reestablishment and protection of wild rhino populations.

## Notes

**Editor**: Ashleigh Donaldson
